# Hereditary Cancer Risk Assessment: Challenges for the Next-Gen Sequencing Era

**DOI:** 10.3389/fonc.2015.00062

**Published:** 2015-03-23

**Authors:** Hugo Pinheiro, Patrícia Oliveira, Carla Oliveira

**Affiliations:** ^1^Instituto de Patologia e Imunologia Molecular da Universidade do Porto (Ipatimup), Porto, Portugal; ^2^Instituto de Investigação e Inovação em Saúde, Universidade do Porto, Porto, Portugal; ^3^Departamento de Patologia e Oncologia, Universidade do Porto, Porto, Portugal

**Keywords:** hereditary cancer, next-gen sequencing, multi-gene panel, whole exome/genome, genetic testing, clinical criteria

The recent advent of next-generation sequencing (NGS) has revolutionized the way we study human diseases in general, and cancer in particular. Novel methodologies and platforms made it possible to resequence, at single nucleotide resolution, entire human exomes, genomes, and epigenomes in a matter of weeks at progressively lower costs. Combined with the possibility of small lab-based setups, this potential leveraged the implementation of such technologies within individual laboratories. This has been of particular importance when numerous genetic tests are required for the evaluation of a given condition, when the condition is genetically heterogeneous, or when genetic testing is not available to confirm a suspected diagnosis (1). In the context of this third scenario, genetic diagnosis has evolved into the implementation of multi-gene sequencing panels, which proved particularly effective for several hereditary conditions (one disease/multiple genes) leading to improvements on the informed genetic testing, prevention, early detection, and management of individuals at high cancer risk (2).

The rationale for the design of multi-gene panels invariably relies on the available knowledge at a certain time. For cancer-associated syndromes, it is common to select the main causative gene(s) known; to add gene(s) causing other syndromes in which the cancer of interest recurrently appears (as part of the tumor spectrum); to identify genes harboring somatic mutations in the cancer of interest, and; eventually, to select genes that cause cancer in other tissues with common embryologic origin. The interpretation of the role of germline mutant genes, when a rationale is present for their selection, is definitely easier than the interpretation of whole exome/genome data, which consistently generates data that surpasses our capacity to draw clinical utility out of it. The whole exome/genome data hold other difficulties, which are commonly referred to as incidental or secondary findings. These “unwanted” and frequently “unexplainable” discoveries depend on the available knowledge, increase the complexity of counseling and management, and are of little use to doctors and patients (3, 4).

A new challenge emerged when germline biallelic mutations in MUTYH were found to greatly increase lifetime risk of colorectal cancer and familial aggregation, raising the awareness for inherited autosomal recessive cancer syndromes (5). Although rare, recessive cancer syndromes may explain the occurrence of cancer aggregation in a proportion of families.

The globalization of NGS infrastructures and the application of this technology, to so far mutation negative families, will also increase the possibility of transforming monogenic diseases into multigenic diseases. It is possible that in some kindred, for which no high penetrance susceptibility gene has been identified, that the combination of germline heterozygotes in lower susceptibility loci, is responsible for the establishment of the phenotype. This possibility would explain lower disease penetrance in some families, variable disease spectrum, and eventually late or early disease onset, if one of the genes acts as a modifier (positive/negative). If factual, this will be soon uncovered with genome-wide approaches.

Overall, NGS will provide, in the near future, a large array of germline mutant genes, which may or may not explain the source of genetic syndromes. The challenge for researchers and clinicians will be to choose the right variant(s) and to prove its role as causative in each individual case. More than ever, combined, and multidisciplinary efforts are required to help deciphering genotype–phenotype correlations in inherited diseases, and to translate NGS data into clinical utility.

There are a few other issues that are worth revisiting when defining cancer-associated syndromes, namely related with the need to disclose histological sub-types of pre-malignant or neoplastic lesions, age of disease onset, and intra-familial disease spectrum.

In several cancer-associated syndromes, histological subtype classification is mandatory to allocate families into syndromes and define the genetic test to be recommended. When pathology reports are missing, clinical criteria may not apply, and the likelihood of families being counseled toward genetic testing decreases. Demonstrating this are morphologically heterogeneous diseases like gastric cancer, for which at least two syndromes have been described (FIGC – familial intestinal gastric cancer and HDGC – hereditary diffuse gastric cancer), which are mainly differentiated based on tumor histo-pathological features (6). As inherited genetic defects have only been found for HDGC (7, 8), all families presenting with aggregation of gastric cancer that do not comply with clinical criteria of HDGC, will not undergo genetic testing. This dependence on pathological data for adequate genetic testing, although useful when single genes were offered in diagnosis, currently limits or prevents the finding of potential causative genetic events, if NGS is applied. So, perhaps genetic testing should not be barred after a given specific pathology diagnosis has been advanced, as it remains unknown whether the established anatomy-based tumor classification is reflected on the genetic level. The finding of a given gene involved in several apparently different cancer-related syndromes, in distinct histological cancer sub-types, or even in distinct organ cancer types will represent a new dimension on the old problem of genotype–phenotype association. This situation is exemplified in a study addressing renal cell carcinoma (RCC) in the context of Cowden syndrome (CS) (9). In this work, the same PTEN germline nonsense mutation was identified in three probands that presented with three different tumor histologies (9). Although the reasons for this remain unknown, it is possible that in CS-RCC, PTEN haplo-insufficiency could cooperate with secondary events (germline or somatic), which may act as genetic modifiers and contribute to promote transformation along distinct histological pathways (9).

We can therefore anticipate that the histological information from a given tumor may even be “disregarded” concerning directing a patient, and their family toward genetic testing (a specific gene/gene panels/full exome). However, histological information should not be overlooked when a genetic defect has been identified, and it is necessary to define a patient’s prognosis and to design adequate surveillance, and treatment strategies.

Early age of disease onset has been recurrently used to define clinical criteria for cancer-associated syndromes, which often restricts testing to those diagnosed with a given cancer under a certain age. However, families presenting with aggregation of cancer, although at later ages of onset than that indicated in clinical criteria, may still present with a germline defect that causes familial aggregation of a given disease. This issue has been elegantly explored in a cohort of Lynch syndrome patients and has opened the way for the relaxation of clinical criteria (10).

Intra-familial disease spectrum is another interesting issue to re-examine in the NGS era, as mutations in cancer genes commonly associated with specific familial disorders, start emerging as the inherited cause in completely different syndromes. This is the case of *CDH1* germline mutations, accepted to cause HDGC, and discovered as the cause of familial breast cancer in families lacking *BRCA* gene mutations (11). Another example is the presence of *TP53* mutations in families with high incidences of gastric cancer, and lacking the classical Li-Fraumeni associated tumor types (12, 13). Actually, *TP53* germline mutations may be involved in many more cases than initially anticipated, as demonstrated by the high frequency of *TP53* germline mutations found in a cohort of unselected cancer patients (14).

All these findings and observations seem to favor the relaxation of clinical criteria for selecting individuals at greater risk of developing familial cancer. In fact, and according to growing evidence, families with aggregation of cancer, independently of age of onset or organ tropism, should be guided for genetic testing, the same way that individuals with very early age of onset, but lacking family history of cancer. Favoring this approach is the fact that a specific syndrome and a specific gene, no longer need to be pinpointed for screening if a multi-gene panel is available. On the other hand, such a strategy may be debatable as the costs of screening may increase, despite the general decrease in price of NGS products and services, due to the large number of patients/families that become eligible for testing with the broadening of clinical criteria.

In summary, the way we have been looking at hereditary cancer risk is changing, and it is urgent to revisit clinical criteria, screening strategies, and clinical management of families with aggregation of cancer in light of the new and still confounding next-gen data, which becomes available on a daily basis.

## Conflict of Interest Statement

The authors declare that the research was conducted in the absence of any commercial or financial relationships that could be construed as a potential conflict of interest.
